# Winding paths to simplicity: genome evolution in facultative insect symbionts

**DOI:** 10.1093/femsre/fuw028

**Published:** 2016-08-12

**Authors:** Wen-Sui Lo, Ya-Yi Huang, Chih-Horng Kuo

**Affiliations:** 1 Institute of Plant and Microbial Biology, Academia Sinica, Taipei 11529, Taiwan; 2 Molecular and Biological Agricultural Sciences Program, Taiwan International Graduate Program, National Chung Hsing University and Academia Sinica, Taipei 11529, Taiwan; 3 Graduate Institute of Biotechnology, National Chung Hsing University, Taichung 40227, Taiwan; 4 Biotechnology Center, National Chung Hsing University, Taichung 40227, Taiwan

**Keywords:** genome degradation, genome size, coding density, horizontal gene transfer, deletional bias, pseudogene, GC content, symbiosis, symbiotic bacteria, *Arsenophonus*, *Hamiltonella*, *Regiella*, *Pantoea*, *Serratia*, *Sodalis*, *Spiroplasma*, *Wolbachia*

## Abstract

Symbiosis between organisms is an important driving force in evolution. Among the diverse relationships described, extensive progress has been made in insect–bacteria symbiosis, which improved our understanding of the genome evolution in host-associated bacteria. Particularly, investigations on several obligate mutualists have pushed the limits of what we know about the minimal genomes for sustaining cellular life. To bridge the gap between those obligate symbionts with extremely reduced genomes and their non-host-restricted ancestors, this review focuses on the recent progress in genome characterization of facultative insect symbionts. Notable cases representing various types and stages of host associations, including those from multiple genera in the family Enterobacteriaceae (class Gammaproteobacteria), *Wolbachia* (Alphaproteobacteria) and *Spiroplasma* (Mollicutes), are discussed. Although several general patterns of genome reduction associated with the adoption of symbiotic relationships could be identified, extensive variation was found among these facultative symbionts. These findings are incorporated into the established conceptual frameworks to develop a more detailed evolutionary model for the discussion of possible trajectories. In summary, transitions from facultative to obligate symbiosis do not appear to be a universal one-way street; switches between hosts and lifestyles (e.g. commensalism, parasitism or mutualism) occur frequently and could be facilitated by horizontal gene transfer.

## Abstract (precis)

This review synthesizes the recent progress in genome characterization of insect-symbiotic bacteria, the emphases include (i) patterns of genome organization, (ii) evolutionary models and trajectories, and (iii) comparisons between facultative and obligate symbionts.

## INTRODUCTION

Symbiosis between organisms is an important driving force in evolution. The most extreme examples involve the formation of mitochondria, which is associated with the origin of eukaryotes (Sagan 1967; Andersson *et al.*^2003^; de Duve 2007; Koonin 2010). Other less intimate associations could still greatly influence the physiology, ecology and evolution of both hosts and their symbionts (Moran 2006; Zilber-Rosenberg and Rosenberg 2008; McFall-Ngai *et al.*^2013^). During the past decade, various insect-symbiont systems have been adopted as models to study how symbiosis impacted the evolution of host-associated bacteria. Particularly, with the rapid development in DNA sequencing technology, biologists could examine the genomes of diverse symbionts, even for those yet to be cultivated outside of their hosts. These genomic investigations of insect symbionts have produced many new and exciting observations. For example, the mealybug *Planococcus citri* (Hemiptera: Pseudococcidae) was found to have a symbiosis system reminiscent of matryoshka dolls, in which the host houses its primary symbiont ‘*Candidatus* Tremblaya princeps’ [the ‘*Candidatus*’ designation denotes the interim taxonomic status (Murray and Stackebrandt 1995); abbreviated as ‘*Ca*.’ below] within specialized cells (i.e. bacteriocytes), while a second symbiont ‘*Ca.* Moranella endobia’ lives intracellularly inside the primary symbiont (McCutcheon and von Dohlen 2011; Husnik *et al.*^2013^; López-Madrigal *et al.*^2013^). Remarkably, genes from the three parties form an interdependent metabolic patchwork to synthesize essential amino acids for supplementing the nutritional deficiency in the host's diet. Undoubtedly, such interactions would have profound influence on all parties involved and these studies advanced our knowledge of biological systems.

In the aforementioned example, as well as other symbiotic bacteria that form obligate associations with their insect hosts, the symbiont genomes were found to be extremely reduced. These symbiont genomes are as small as 0.1–0.2 million base pairs (Mb) in size and contain only 100–200 coding DNA sequences (CDSs), which are simpler than some organelles and viruses (McCutcheon and Moran 2012). As such, studies on these symbionts push the limit of what we know about the minimal gene set required for sustaining cellular life (Maniloff 1996; Koonin 2000, 2003), as well as provide insights into the evolution of organelles (Brown 2003; Toft and Andersson 2010; Keeling, McCutcheon and Doolittle 2015). With the extensive research attention given to these obligate insect symbionts, multiple high profile reviews have been published in recent years to synthesize the progress made (Moran, McCutcheon and Nakabachi 2008; Moya *et al.*^2008^; McCutcheon 2010; Oliver *et al.*^2010^; Shigenobu and Wilson 2011; McCutcheon and Moran 2012; Wernegreen 2012; Moran and Bennett 2014; Bennett and Moran 2015). However, the genome evolution in facultative insect symbionts, defined as those not required for host survival and usually have partial infection rates among host populations, has also made considerable progress but received much less attention in reviews. To fill this gap, this review focuses on the recent progress in genome characterization of facultative insect symbionts. Specifically, the emphases include (i) patterns of genome organization, (ii) evolutionary models and trajectories, and (iii) comparisons between facultative and obligate symbionts. The taxonomic groups discussed include multiple genera in the family Enterobacteriaceae (class Gammaproteobacteria), *Wolbachia* (Alphaproteobacteria) and *Spiroplasma* (Mollicutes). Special attention is given to *Spiroplasma* because this genus is poorly covered in previous reviews on bacteria genome evolution, yet has experienced a surge of genomic studies in recent years and is a major study system of our research group. In contrast, bacteria groups that are primarily insect-transmitted pathogens of vertebrates or plants such as *Rickettsia* (Gillespie *et al.*^2008^) or ‘*Ca.* Phytoplasma’ (Hogenhout *et al.*^2008^) are purposely omitted in this review. The main reason is that the patterns of evolution in those groups are expected to be greatly influenced by the interactions with their vertebrate and plant hosts, which is a complex issue outside of our scope.

## PREVIOUS OBSERVATIONS AND ESTABLISHED FRAMEWORKS

**Figure 1.**
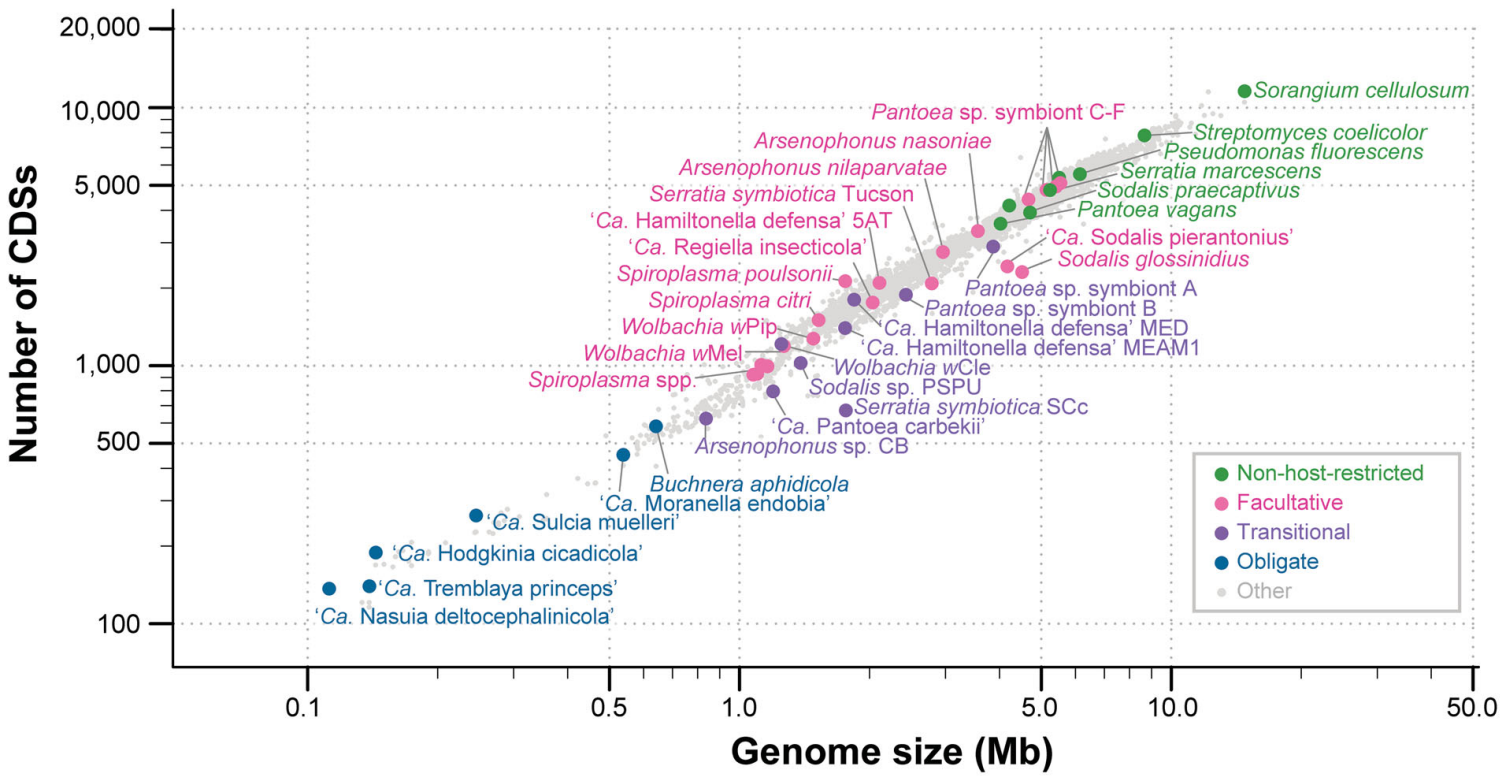
Association between genome size and the number of CDSs in bacteria. Based on the 5043 complete genomes available from GenBank as of March 2016 and additional genomes listed in Table 1. Representative lineages are color-coded by the lifestyle.

Among all bacterial genomes that have been characterized to date, the genome sizes span approximately two orders of magnitude (Fig. 1 and Table 1). The largest one was found in a soil-dwelling bacterium *Sorangium cellulosum* (Han *et al.*^2013^), which has a chromosome that is 14.8 Mb in size and contains 11 599 CDSs. The smallest one was found in an obligate insect symbiont ‘*Ca*. Nasuia deltocephalinicola’ (Bennett and Moran 2013), which has a 0.112 Mb chromosome with 137 CDSs. In bacteria, the genome size is important because this feature is strongly correlated with the number of CDSs (Lynch 2006; Kuo, Moran and Ochman 2009; Burke and Moran 2011), the complexity of metabolic pathways as well as the ecological niches occupied (Ochman and Davalos 2006; Kuo, Moran and Ochman 2009; Toft and Andersson 2010; McCutcheon and Moran 2012; Land *et al.*^2015^).

**Table 1.** Genome size, GC content, coding density and the number of CDSs found among representative bacteria lineages.

Organism	Lifestyle^a^	Genome size (bp)	GC content (%)	Coding density (%)	No. of CDSs	References
**Non-host-restricted bacteria**						
*Sorangium cellulosum*	N	14 782 125	72.1	89.2	11 599	Han *et al.* (2013)
*Streptomyces coelicolor*	N	8667 507	72.1	88.9	7825	Bentley *et al.* (2002)
*Pseudomonas fluorescens*	N	6143 950	60.4	89.5	5534	Cho *et al.* (2015)
*Escherichia coli*	N	5498 450	50.5	88.1	5361	Hayashi *et al.* (2001)
*Bacillus subtilis*	N	4215 606	43.5	87.6	4185	Kunst *et al.* (1997)
**Facultative symbionts and their congeners**						
*Pantoea vagans*	N	4024 986	55.5	86.4	3556	Smits *et al.* (2010)
*Pantoea* sp. symbiont A	T	3869 537	57.0	N/A	2900	Hosokawa *et al.* (2016)
*Pantoea* sp. symbiont B	T	2429 186	56.6	N/A	1886	Hosokawa *et al.* (2016)
*Pantoea* sp. symbiont C	F	5141 071	58.9	N/A	4812	Hosokawa *et al.* (2016)
*Pantoea* sp. symbiont D	F	5537 063	54.1	N/A	5113	Hosokawa *et al.* (2016)
*Pantoea* sp. symbiont E	F	5409 024	54.2	N/A	4959	Hosokawa *et al.* (2016)
*Pantoea* sp. symbiont F	F	4670 903	57.4	N/A	4412	Hosokawa *et al.* (2016)
‘*Ca*. Pantoea carbekii’	T	1197 048	30.6	67.4	797	Kenyon, Meulia and Sabree (2015)
*Serratia marcescens*	N	5241 455	59.6	87.1	4806	Chung *et al.* (2013)
*Serratia symbiotica* Tucson	F	2789 218	52.0	60.9	2089	Burke and Moran (2011)
*Serratia symbiotica* SCc	T	1762 765	29.2	38.7	672	Lamelas *et al.* (2011)
*Sodalis praecaptivus*	N	4709 528	57.5	81.9	3933	Oakeson *et al.* (2014)
‘*Ca*. Sodalis pierantonius’	F	4513 140	56.1	46.2	2309	Oakeson *et al.* (2014)
*Sodalis glossinidius*	F	4171 146	54.7	50.9	2432	Toh *et al.* (2006)
*Sodalis* sp. PSPU	T	1386 675	54.2	N/A	1027	Koga and Moran (2014)
‘*Ca*. Hamiltonella defensa’ 5AT	F	2110 331	40.1	80.8	2100	Degnan *et al.* (2009)
‘*Ca*. Hamiltonella defensa’ MED	T	1800 792	40.5	84.4	1884	Rao *et al.* (2015)
‘*Ca*. Hamiltonella defensa’ MEAM1	T	1726 317	40.1	78.8	1400	Rollat-Farnier *et al.* (2015)
‘*Ca*. Regiella insecticola’	F	2035 106	42.4	71.4	1761	Degnan *et al.* (2010)
*Arsenophonus nasoniae*	F	3567 128	37.4	73.2	3332	Darby *et al.* (2010); Wilkes *et al.* (2010)
*Arsenophonus nilaparvatae*	F	2960 000	37.6	83.1	2762	Xue *et al.* (2014)
‘*Ca*. Arsenophonus melophagi’	T	1155 312	32.2	61.3	725	Nováková *et al.* (2015)
*Arsenophonus* sp. CB	T	836 724	24.0	74.7	625	GenBank: CP013920
*Wolbachia w*Pip	F	1482 455	34.2	81.7	1275	Klasson *et al.* (2008)
*Wolbachia w*Mel	F	1267 782	35.2	80.0	1195	Wu *et al.* (2004)
*Wolbachia w*Cle	T	1250 060	36.3	74.6	1216	Nikoh *et al.* (2014)
*Spiroplasma poulsonii*	F	1757 846	26.5	76.6	2129	Paredes *et al.* (2015)
*Spiroplasma citri*	F	1525 756	26.1	74.0	1504	Carle *et al.* (2010)
*Spiroplasma eriocheiris*	F	1365 714	29.8	86.0	1180	Lo, Gasparich and Kuo (2015)
*Spiroplasma culicicola*	F	1175 131	26.4	92.2	1071	Chang *et al.* (2014)
*Spiroplasma apis*	F	1160 554	28.3	87.8	997	Ku *et al.* (2014)
*Spiroplasma atrichopogonis*	F	1160 484	29.3	71.5	996	Lo, Gasparich and Kuo (2015)
*Spiroplasma chrysopicola*	F	1123 322	28.8	89.0	1009	Ku *et al.* (2013)
*Spiroplasma syrphidicola*	F	1107 344	29.2	90.4	1006	Ku *et al.* (2013)
*Spiroplasma melliferum*	F	1098 846	27.5	85.1	932	Lo *et al.* (2013a)
*Spiroplasma sabaudiense*	F	1075 953	30.2	90.0	924	Chang *et al.* (2014)
*Spiroplasma taiwanense*	F	1075 140	23.9	82.5	991	Lo *et al.* (2013b)
*Spiroplasma diminutum*	F	945 296	25.5	92.7	858	Lo *et al.* (2013b)
**Obligate mutualists with highly reduced genomes**						
*Buchnera aphidicola*	O	640 681	26.3	88.0	583	Shigenobu *et al.* (2000)
‘*Ca*. Moranella endobia’	O	538 294	43.5	79.0	452	McCutcheon and von Dohlen (2011)
‘*Ca*. Sulcia muelleri’	O	245 530	22.4	96.1	263	McCutcheon and Moran (2007)
‘*Ca*. Hodgkinia cicadicola’	O	143 795	58.4	95.1	189	McCutcheon, McDonald and Moran (2009)
‘*Ca*. Tremblaya princeps’	O	138 927	58.8	72.9	140	McCutcheon and von Dohlen (2011)
‘*Ca*. Nasuia deltocephalinicola’	O	112 091	17.1	91.4	137	Bennett and Moran (2013)

a Abbreviations: (N)on-host-restricted; (F)acultative; (T)ransitional; (O)bligate.

Through phylogenetic character mapping and other evolutionary analyses, it is well established that the present-day symbionts have evolved from free-living ancestors not dependent on eukaryotic hosts (Moran and Plague 2004; Ochman 2005; Moran, McCutcheon and Nakabachi 2008; Toft and Andersson 2010; McCutcheon and Moran 2012). In addition to the reduction in overall genome size and the loss of CDSs, other changes occurred during this evolutionary process include acceleration in mutation accumulation rates (Kuo and Ochman 2009b; Toft and Andersson 2010; McCutcheon and Moran 2012) and increases in protein functional complexity (Kelkar and Ochman 2013).

One hypothesis to explain the genome reduction universally observed in symbionts, as proposed by Kuo, Moran and Ochman (2009), was constructed based on the following observations and logic. First, because most bacteria experienced strong biases toward deletions in their mutational input (Andersson and Andersson 2001; Mira, Ochman and Moran 2001; Nilsson *et al.*^2005^; Kuo and Ochman 2009a), chromosomal segments that do not contribute to fitness are unlikely to persist, even in the absence of selection. Due to the deletional bias, most bacterial genomes have a very high coding density, with 85%–90% of the chromosome being protein-coding regions (Lynch 2006; Kuo, Moran and Ochman 2009; McCutcheon and Moran 2012). Compared to free-living bacteria, host-associated bacteria typically exhibit elevated levels of genetic drift (Hershberg, Tang and Petrov 2007; Kuo, Moran and Ochman 2009; Novichkov *et al.*^2009^), presumably because the dependence on eukaryotic hosts for survival and reproduction reduces their effective population size. The elevation in genetic drift increases the fixation probability of slightly deleterious mutations, which are likely to be deletions that convert full-length CDSs into pseudogenes and reduce chromosome size. Additionally, invasion and proliferation of selfish genetic elements (Ochman and Davalos 2006), loss of DNA repair genes (Moran, McCutcheon and Nakabachi 2008) and replication slippage in homopolymeric runs (Moran, McLaughlin and Sorek 2009) all contributed to the pseudogenization events observed in symbiont genomes. Because pseudogenes may be slightly deleterious (Kuo and Ochman 2010), deletions that remove pseudogenes may be favored by positive selection, leading to further acceleration in genome reduction. Additionally, in becoming a symbiont, a large number of genes that were once essential in the free-living ancestor (e.g. biosynthesis pathways for nutrients available from the hosts) would be released from selective constraint, allowing these chromosomal regions to be removed through mutation accumulations, even if this process of genome reduction is not necessarily adaptive.

A decade ago, when the information on bacterial genomes was more limited, a simple scheme was proposed to classify bacteria based on their lifestyle and genome size (Ochman and Davalos 2006). The three categories include (i) free-living bacteria with large genomes (5–10 Mb), (ii) recent or facultative pathogens with intermediate genomes (2–5 Mb) and (iii) obligate symbionts or pathogens with small genome (0.5–1.5 Mb). As the knowledge of symbiont genome and biology improved, a more refined classification was introduced to describe the obligate and facultative symbionts (Moran, McCutcheon and Nakabachi 2008). In this classification, obligate symbionts are defined as those with a long coevolutionary history with the host, residing intracellularly in special host organ (i.e. bacteriome) to provide the host with nutrients, and have highly reduced genomes (<1 Mb) that are stable from gene acquisitions, mobile genetic element invasions and genome rearrangements. For facultative symbionts, three subcategories are defined: (i) mutualists that benefit hosts but are not essential, (ii) reproductive manipulators that selfishly distort the sex ratio of host progeny (e.g. male killing to increase vertical transmission through females) and (iii) symbionts with unknown effect. The genomes of these facultative symbionts typically have experienced moderate gene inactivation, may be highly dynamic with chromosome rearrangements and proliferation of mobile genetic elements, and have a chromosome size of >1 Mb. More recently, with the discovery of symbionts with extremely reduced genomes, a four-category scheme was proposed (McCutcheon and Moran 2012), which includes (i) free-living/non-host-restricted bacteria, (ii) recently host-restricted symbionts or pathogens (2–4 Mb), (iii) long-term obligate symbionts or pathogens (0.4–0.7 Mb) and (iv) tiny-genome symbionts (0.1–0.2 Mb). Later, a more detailed review was devoted to those tiny-genome symbionts (Moran and Bennett 2014). To incorporate more recent findings and improve upon these schemes, notable examples of recently evolved facultative insect symbionts are discussed in further details below.

## RECENT PROGRESS IN FACULTATIVE INSECT SYMBIONTS

### Overview

In the following subsections, notable examples of facultative insect symbionts, as well as those that may be transitional in between facultative and obligate relationships with their hosts, are organized by genus (Table 1). With the exception of *Wolbachia* and *Spiroplasma*, all other genera belong to the family Enterobacteriaceae within the class Gammaproteobacteria. Recent progress in understanding their genome evolution, particularly observations relating to genome reduction and gene loss, is discussed. When possible, lineages belonging to the same genus that are non-host-restricted and obligate symbionts are discussed as references for the ancestral state and possible evolutionary trajectories. To provide context, the ecology, phylogeny and other aspects of symbiont biology are briefly discussed as well.

### Pantoea

The genus *Pantoea* contains 20 named species with diverse ecological niches (Walterson and Stavrinides 2015), including those described as strictly environmental (e.g. *Pantoea gavinae*), plant-associated (e.g. *P. anthophila*, *P. cypripedii* and *P. rodasii*) or clinical (e.g. *P. brenneri*, *P. eucrina*, and *P. septica*). However, other species have been found in multiple niches, suggesting that the transition between niches may not be difficult for these bacteria. Current available information indicates that these bacteria have a genome size of 4.5–6.3 Mb with a GC content of 52%–55% (Walterson and Stavrinides 2015), which is typical in Enterobacteriaceae.

In addition to these named species, there are multiple unnamed lineages that have successfully established symbiotic relationships with stink bugs (Prado and Almeida 2008; Hosokawa *et al.*2010a, 2016; Bansal, Michel and Sabree 2014). One recent study on this system revealed that there are six *Pantoea* lineages (termed symbionts A–F) found in the brown-winged green stink bug *Plautia stali* (Hemiptera: Pentatomidae) in Japan (Hosokawa *et al.*^2016^). Because the symbionts found among different host populations exhibit extensive geographical variation, these associations appear to have formed recently. Moreover, the molecular phylogeny indicated that these six lineages fall in at least three strongly supported clades, suggesting that these associations are likely to have developed multiple times within the genus. Although the mechanism is unknown, disrupting the vertical transmission of symbionts via egg surface sterilization prevents nymphs from reaching adulthood, supporting that these associations are essential for host survival. However, the growth defect could be rescued by exposing the symbiont-free nymphs to soil samples collected from different habitats. From these rescued adults, symbionts C, D or E were found to have colonized the midgut, indicating that these bacteria are facultative symbionts that could persist in soil environments outside of the host and are promiscuous in terms of the host genotype. Laboratory culture also showed that symbionts C, D, E and F are readily cultivable on standard Luria–Bertani agar plates. In contrast, symbionts A and B could not be re-acquired from environment and are not cultivable yet, suggesting that these two lineages may be transitioning into obligate associations with their hosts. Intriguingly, the initial genome survey found a pattern that is consistent with this hypothesis and the general predictions of symbiont genome evolution (Table 1). Symbionts C–F have genome characteristics similar to other non-host-restricted *Pantoea* (genome size = 4.7–5.5 Mb, containing 4412–5113 CDSs), while symbionts A and B both have a much more reduced genome (symbiont A: 3.9 Mb with 2900 CDSs; symbiont B: 2.4 Mb with 1886 CDSs). Unfortunately, a more detailed comparative genomics analysis is not available so it is unclear what are the genetic differentiations between A/B and C–F. Future studies on these symbiont genomes, as well as comparisons with other non-host-restricted species within *Pantoea*, may provide important insights into the genomic changes associated with transitions between lifestyles.

**Figure 2.**
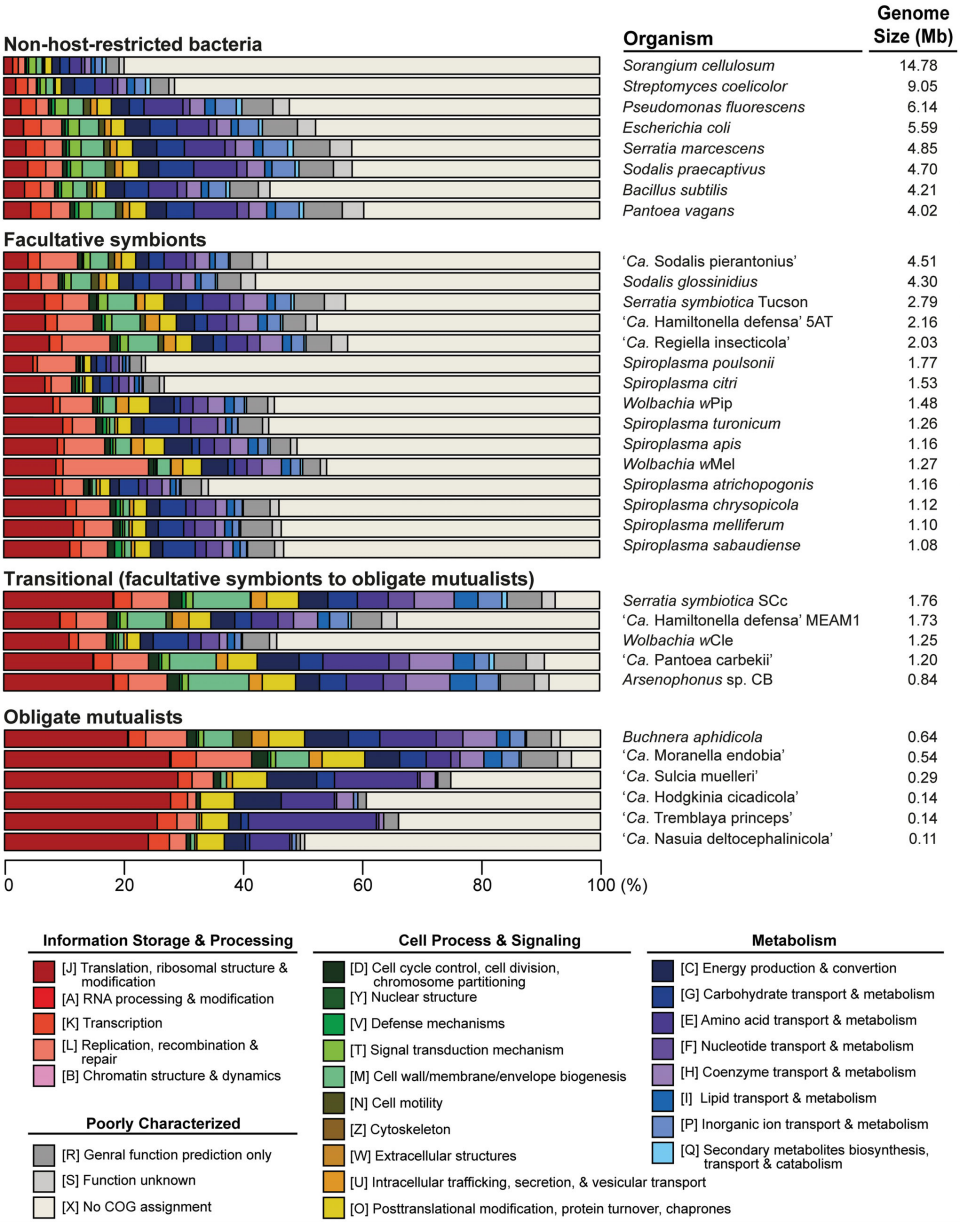
Functional classification of protein-coding genes. Functional category assignments are based on the COG database (Tatusov, Koonin and Lipman 1997; Tatusov *et al.*^2003^), the procedure of data analysis is based on that described in Lo *et al.* (2013a). Not all genomes listed in Table 1 are included for the following reasons: for closely related *Spiroplasma* species with nearly identical patterns, only few representatives are selected; for other genera, several draft genomes are omitted because the sequence records obtained from GenBank lack annotation of genes.

A second example of *Pantoea*-stinkbug symbiosis was found between ‘*Ca*. Pantoea carbekii’ and the brown marmorated stink bug *Halyomorpha halys* (Hemiptera: Pentatomidae) in North America (Bansal, Michel and Sabree 2014). In this case, the same symbiont lineage was found in all populations surveyed, possibly because the host is a recently introduced invasive species that spread across North America since mid-1990. Similar to the previous example, the symbiont colonizes the host midgut crypts and is vertically transmitted via egg surface contamination (Kenyon, Meulia and Sabree 2015), and symbiont removal by egg sterilization greatly impacts the host fitness (Taylor *et al.*^2014^). The symbiont genome is strikingly small, with the chromosome being 1.2 Mb in size and contains 797 CDSs (Kenyon, Meulia and Sabree 2015). These measurements of genome size indicate that this symbiont has lost >70% of the chromosomal segments and protein-coding genes compared to its non-host-restricted relatives. Moreover, the GC content is reduced to 30.6%, a pattern that is commonly observed among symbiotic bacteria with highly reduced genomes (Table 1). In terms of gene content, several genes related to DNA replication and repair are missing (e.g. *phr*, *xth* and *rep*) or disrupted by frameshift mutations and premature stop codons (e.g. *polA* and *ligA*), which is consistent with the elevated mutation accumulation rate observed. Additionally, genes related to cell divisions (e.g. *ftsK* and *ftsN*) are degraded, which may be linked to the non-uniform cell morphologies observed. These findings are parallel to the patterns found among obligate symbionts with highly reduced genomes (McCutcheon and Moran 2012) and the overall gene functional category distribution of this bacterium is similar to those found in obligate mutualists (Fig. 2). However, unlike some intracellular obligate symbionts that have lost their cell wall (e.g. ‘*Ca*. Carsonella ruddii’, ‘*Ca*. Hodgkinia cicadicola’, ‘*Ca*. Tremblaya princeps’ and ‘*Ca*. Zinderia insecticola’, but not the ancient *Buchnera*), the ‘*Ca*. Pantoea carbekii’ genome still retains the genes for peptidoglycan production, possibly for its survival on the surface of host eggs. Intriguingly, despite the massive gene loss, genes involved in biosynthesis of amino acids, vitamins and cofactors appeared to have been selectively retained. Furthermore, its four plasmids contain genes important for nitrogen assimilation and thiamine biosynthesis. Taken together, these patterns suggest that ‘*Ca*. Pantoea carbekii’ may be on an evolutionary trajectory of becoming a nutritional mutualist for its phytophagous host.

### Serratia

*Serratia* is a large and diverse genus containing species described as environmental, plant-associated or opportunistic pathogens (Garbeva, van Elsas and de Boer 2012; Devi *et al.*^2013^; Kuo *et al.*^2013^; Müller *et al.*^2013^; Petersen and Tisa 2013; Iguchi *et al.*^2014^; Bonnin *et al.*^2015^; Jeong, Kloepper and Ryu 2015). Two species with notable insect associations include a highly virulent insect pathogen *Serratia liquefaciens* (Egami *et al.*^2009^) and *S. nematodiphila*, which is a mutualistic symbiont of entomopathogenic nematodes (Zhang *et al.*^2009^; Abebe *et al.*^2011^). However, genome characterization of these two species revealed that their genome characteristics are similar to those non-host-restricted *Serratia*, with a chromosome that is >5 Mb in size, 55%–60% GC and containing >4500 CDSs (Taira *et al.*^2014^; Kwak, Khan and Shin 2015).

Currently, one formally described species within this genus, *S. symbiotica*, is known for its endosymbiotic relationship with multiple aphid subfamilies (Moran *et al.*^2005^; Lamelas *et al.*^2008^; Sabri *et al.*^2011^). However, strains assigned to this species belong to at least two clades, each with distinct characteristics. Clade A strains are associated with the aphid subfamily Aphidoidea and are facultative symbionts that protect their hosts from heat stress (Burke, Fiehn and Moran 2009). Although this symbiont is preferentially found intracellularly in host cells adjacent to primary bacteriocytes (which harbor the primary nutritional mutualist *Buchnera aphidicola*), it also exists extracellularly in the hemolymph (Moran *et al.*^2005^). In comparison, clade B strains are associated with the aphid subfamily Lachninae, and appear to be in transition into an obligate endosymbiont with a change in cell morphology and restriction to specific bacteriocytes (Lamelas *et al.*^2008^). Recent genome characterization of these strains provided genetic explanations to these morphological and biological observations.

One clade A representative, *S. symbiotica* Tucson sampled from the pea aphids *Acyrthosiphon pisum*, was found to have a moderately reduced genome (Burke and Moran 2011). The 16S rRNA gene of this strain shows 97%–98% sequence identity to non-host-restricted *Serratia* lineages, suggesting that the divergence has occurred recently. The third codon positions have a GC content of 63% compared to 68% in *S. proteamaculans* and 79% in *S. marcescens*, indicating accumulation of GC to AT mutations. Moreover, the rate of non-synonymous substitution is twice as high as other *Serratia*. Both findings are consistent with the general observations of symbiont genome evolution. Notably, the genome size has decreased to 2.8 Mb, which is 60% of its non-host-restricted congeners. This strain appears to be experiencing an ongoing process of large-scale genome degradation, with its chromosome having a coding density of 60.9%, 2098 intact CDSs and 550 pseudogenes. Presumably, the deletional bias observed in bacteria genomes would remove these pseudogenes in the near future, leading to further reduction in genome size. In terms of gene function, many biosynthesis pathways (e.g. essential amino acids, nucleotides and cofactors) have degraded, suggesting that this bacterium must rely on its insect host, or more likely, the primary symbiont *Buchnera* for these nutrients. The losses of biosynthesis pathways for various nutrients are commonly observed in host-dependent bacteria, including mutualists and pathogens. However, in this clade A representative of *S. symbiotica*, several genes linked to bacterial pathogenesis (e.g. iron acquisition and type IV pili for attachment to host cells) have been pseudogenized, suggesting that it is not on an evolutionary trajectory of becoming a pathogen.

In comparison, genome analysis of a clade B representative *S. symbiotica* SCc from the aphid *Cinara cedri* suggests that it has become an integral part of the obligate tripartite mutualism (Lamelas *et al.*^2011^). This strain is further down the path of genome reduction, with a chromosome size of 1.7 Mb and a GC content of 29.2%. Surprisingly, despite having only 672 CDSs, these genes fully complement the gene losses specific to the primary symbiont *Buchnera* sharing the same host (i.e. the *Buchnera* in the pea aphid lineage still possesses these genes), such that these two symbionts could synthesize several essential amino acids (tryptophan and lysine) and cofactors (biotin, folate and coenzyme A) through their interdependent metabolic network.

### Sodalis

The genus *Sodalis* includes two lineages that appear to be in early stages of the massive genome degradation associated with becoming vertically transmitted endosymbionts (Toh *et al.*^2006^; Clayton *et al.*^2012^; Oakeson *et al.*^2014^) and one that has become an obligate coprimary mutualist with a highly reduced genome (Koga and Moran 2014). Additionally, several other insect-symbiotic lineages have been identified but their genomes are yet to be characterized (Kaiwa *et al.*^2010^; Chrudimský et al. 2012; Toju *et al.*^2013^; Hosokawa *et al.*^2015^).

Other than these symbionts, one notable recent finding within this genus is an accidental discovery of a non-host-restricted lineage, which served as a valuable reference of the ancestral state to infer genome evolution (Clayton *et al.*^2012^; Oakeson *et al.*^2014^). This particular lineage, *Sodalis praecaptivus*, was described as an opportunistic human pathogen, isolated from the cyst of a patient who suffered a puncture wound from crab apple tree branches (Clayton *et al.*^2012^). Genome survey revealed that the strain is closely related to those symbionts and has a chromosome typical among non-host-restricted Enterobacteriaceae (i.e. 4.7 Mb in size, 57.5% GC, 81.9% coding, 3993 intact CDSs and 61 pseudogenes) (Oakeson *et al.*^2014^).

In comparison, the two recently evolved symbionts, *So. glossinidius* from the tsetse flies *Glossina* (Diptera: Glossinidae) (Dale and Maudlin 1999; Toh *et al.*^2006^) and ‘*Ca.* Sodalis pierantonius’ from the rice weevil *Sitophilus oryzae* (Coleoptera: Curculionidae) (Clayton *et al.*^2012^), both have a chromosome that is 89%–96% in size yet containing only 59%–62% of the genes compared to their non-host-restricted relative *So. praecaptivus* (Table 1). Interestingly, examination of gene inactivation events revealed multiple homologous genes that were lost independently in these two genomes through accumulation of different mutations. Moreover, while two degraded genomes differ substantially in their exact gene content (i.e. only 1229 orthologous genes are shared through comparisons to *So. praecaptivus*), the overall functional category distribution remains highly similar (Clayton *et al.*^2012^; Oakeson *et al.*^2014^). One possible explanation to these observations is that the genes released from selective constraint during the transition to endosymbiosis could be similar in both lineages. However, because the massive genome degradation is mainly driven by stochastic events of mutation accumulation, the exact genes lost since the divergence could vary substantially between closely related lineages. Nonetheless, due to the similarity in overall physiology, gene content comparison based on broad functional categories is expected to generate similar overall patterns (Fig. 2).

In contrast to the aforementioned *S. symbiotica* SCc system (Lamelas *et al.*^2011^), where a coprimary symbiont was newly acquired to complement the irreversible gene loss occurred in the primary symbiont, the *Sodalis* lineages in spittlebugs (Hemiptera: Aphrophoridae) represent a rare case of coprimary symbiont replacement (Koga *et al.*^2013^). In this system, the ancestral host had a coprimary symbiont ‘*Ca*. Zinderia insecticola’ (Betaproteobacteria) to complement the gene loss in the primary symbiont ‘*Ca*. Sulcia muelleri’ (Bacteroidetes), thus maintaining an obligate tripartite nutritional symbiosis. However, in the common ancestor of the tribe Philaenini, the ‘*Ca*. Zinderia’ was replaced by a *Sodalis*. The strain *Sodalis* sp. PSPU from the meadow spittlebug *Philaenus spumarius* (Hemiptera: Aphrophoridae) was found to have a genome of 1.4 Mb with 1027 CDSs (Koga and Moran 2014). Although small compared to other *Sodalis*, this genome is much larger and more complex than the present-day ‘*Ca*. Zinderia’ in other spittlebugs (0.2 Mb with 202 CDSs) (McCutcheon and Moran 2010). Importantly, it retains the genes required to complement the amino acids biosynthesis pathways in the coexisting ‘*Ca*. Sulcia’, as well as provides some redundancy in these crucial pathways of maintaining the symbiosis. With time, these redundant genes and pathways may be lost through mutation accumulation as observed in other systems involving two obligate mutualists. Compared to the ‘*Ca*. Zinderia’ found in other spittlebugs, this *Sodalis* contains several genes for efficient energy production, which presumably relaxes the severe energy limitation of its xylem-feeding host.

These cases of coprimary symbiont acquisitions have an important evolutionary implication. For an obligate endosymbiont, gene loss through mutation accumulations could lead to failure of the symbiosis, which would result in extinction of the symbiont and the host. The possibility of acquiring new symbionts provides the host a way to circumvent the Muller's ratchet operating on its old partner (Moran 1996), or even further expand its ecological niches.

### ‘Ca. Hamiltonella’ and ‘*Ca*. Regiella’

‘*Ca.* Hamiltonella defensa’ and ‘*Ca.* Regiella insecticola’ are two closely related facultative symbionts commonly found in aphids (Moran *et al.*^2005^). The former protects the host from parasitoid wasps (Oliver *et al.*^2003^), while the latter helps to resist fungal infection (Scarborough, Ferrari and Godfray 2005). Similar to *S. symbiotica*, these two bacteria exist both intraceullarly and extracellularly inside their hosts (Moran *et al.*^2005^; Tsuchida *et al.*^2005^). In addition to vertical transmission through female ovaries, these symbionts could also be sexually transmitted through male accessary glands (Moran and Dunbar 2006). Comparative genomics analysis between ‘*Ca*. Hamiltonella defensa’ 5AT and ‘*Ca.* Regiella insecticola’ LSR1, both from the pea aphids *A. pisum*, reveals that these two strains are similar in their overall genome size and GC content (Degnan *et al.*^2009^, ^2010^). The genome of this ‘*Ca.* Regiella’ is slightly more degraded, with a coding density of 71.4% and 1761 intact CDSs. In comparison, ‘*Ca*. Hamiltonella defensa’ 5AT has a coding density of 80.8% and 2100 intact CDSs (Table 1). However, despite these similarities and a close relationship inferred from their molecular phylogeny, the two genomes share only 918 single-copy genes and have very little conservation in gene order. This pattern of gene content divergence between closely related species, presumably through differential gene loss mainly driven by stochastic events, is similar to that found between *So. glossinidius* and ‘*Ca.* Sodalis pierantonius’. However, in these two genomes, most of the pseudogenes have been lost as well, resulting in much smaller genome sizes while the coding densities are more similar to other enterobacteria. In terms of functions, both genomes have maintained the biosynthesis pathways for two essential amino acids (i.e. threonine and lysine) and several cofactors (i.e. coenzyme A, isoprenoids, ubiquinone and vitamin B_2_/B_3_/B_6_/B_9_). It is unclear if this observation was shaped by selection.

The evolution of ‘*Ca*. Hamiltonella defensa’ has another similarity to *S. symbiotica* in that the lineages associated with different hosts have adopted different functional roles, leading to different patterns of genome reduction. While the aphid-associated strains of ‘*Ca*. Hamiltonella defensa’ are facultative defensive symbionts and their infection frequencies are correlated with parasitoid pressure (Oliver *et al.*^2008^), the ‘*Ca*. Hamiltonella defensa’ associated with the whiteflies *Bemisia tabaci* species complex (Hemiptera: Aleyrodidae) have become coprimary nutritional mutualists. Through genome analysis of two whitefly associated strains, MED (Rao *et al.*^2012^, ^2015^) and MEAM1 (Rollat-Farnier *et al.*^2015^), it was found that these whitefly associated ‘*Ca*. Hamiltonella defensa’ have more reduced genomes (Table 1). The two type III secretion systems found in the aphid-associated 5AT, which are assumed to be required for invading new hosts during its horizontal transmission, are lost in both of the whitefly associated strains. Additionally, the phage-originated toxin genes for the protection against parasitoids (Oliver *et al.*^2009^) have been lost as well. However, genes involved in cysteine and lysine biosynthesis that are absent in 5AT have been maintained in both MED and MEAM1. These genes complement the gene loss in the whitefly primary nutritional mutualist *Portiera.* These results from the genome analysis, as well as other lines of evidence such as their infection frequency (Gueguen *et al.*^2010^; Pan *et al.*^2012^) and their coresidence with *Portiera* in the primary bacteriocytes (Rao *et al.*^2015^), all support that these whitefly associated ‘*Ca*. Hamiltonella defensa’ have provided yet another example of evolutionary transition from facultative symbionts to obligate mutualists.

### Arsenophonus

The genus *Arsenophonus* contains diverse lineages found in a wide range of insects (Nováková, Hypša and Moran 2009; Jousselin *et al.*^2013^). Although their ecology remains to be better characterized, available case reports indicate that several types of symbiosis have evolved among these endosymbionts. The first described species *Arsenophonus nasoniae* is a male-killing reproductive parasite of the parasitoid wasps *Nasonia vitripennis* (Hymenoptera: Pteromalidae) (Gherna *et al.*^1991^). Notably, this bacterium has a lifecycle that is highly unusual compared to other more well-known reproductive parasites such as *Wolbachia*. Instead of direct vertical transmission through female ovaries to eggs, the bacteria are injected into the prey during oviposition, ingested by the feeding wasp larvae, and then invade through the wasp larval gut to establish infection in the next generation (Werren, Skinner and Huger 1986). In other words, this bacterium utilizes two alternative hosts and is capable of both vertical and horizontal transmission through this complex lifecycle. Genome analysis of this bacterium revealed its chromosome is 3.6 Mb in size and contains 3332 CDSs (Darby *et al.*^2010^; Wilkes *et al.*^2010^), which is only moderately reduced compared to other non-host-restricted Enterobacteriaceae and 2–3X of *Wolbachia* (Table 1). As expected from its complex lifecycle, diverse arrays of arsenal for infection (e.g. multiple type III secretion systems, toxins, adhesins, etc) are found in its gene content. However, biochemical pathways associated with mutualism (e.g. amino acid biosynthesis) are mostly disrupted or entirely absent, suggesting specialization of a parasitic lifestyle. Interestingly, multiple putative horizontal gene acquisitions, including one potentially from *Wolbachia*, have been identified (Darby *et al.*^2010^).

Although the direct evidence is still lacking, it is possible that some *Arsenophonus* lineages have become obligate mutualists. In the brown planthopper *Nilaparvata lugens* (Hemiptera: Delphacidae), the insect host relies on a filamentous ascomycete fungus in its fat body as the primary symbiont for nutritional provisioning (Xue *et al.*^2014^). The fungal symbiont could provide all essential amino acids, nitrogen recycling and steroid biosynthesis, but it is deficient in several vitamin biosynthesis pathways. Genome sequencing of the *Ar. nilaparvatae* from the same host indicates that this bacterium has a complete gene set for B vitamin synthesis, suggesting that it may act as a nutritional mutualist. The *Ar. nilaparvatae* genome is slightly smaller than the parasitic *Ar. nasoniae* (Table 1) and it is unclear if these vitamin synthesis genes were retained from the ancestor or acquired horizontally. Intriguingly, these brown planthoppers are always infected with either the *Ar. nilaparvatae* or an uncharacterized *Wolbachia* (Qu *et al.*^2013^). Because a *Wolbachia w*Cle has been found to become an essential mutualist for providing B vitamins to its bedbug hosts (Hosokawa *et al.*2010b; Nikoh *et al.*^2014^), it is possible that the *Ar. nilaparvatae* and this uncharacterized *Wolbachia* are competitors for the same niche in these brown planthoppers (Xue *et al.*^2014^).

In the case of the ‘*Ca.* Arsenophonus melophagi’ from the sheep keds, *Melophagus ovinus* (Diptera: Melophagus), the symbiont is present in all examined adult hosts, existing intracellularly in specialized cells of the host intestine wall and extracellularly in the lumen of the milk glands, suggesting that it could establish stable associations and be vertically transmitted (Nováková *et al.*^2015^). Preliminary genome survey revealed that this genome is much more reduced compared to the two other *Arsenophonus* lineages above (Table 1). The genome characteristics were described as being similar to the primary obligate nutritional mutualist *Wigglesworthia* found in tsetse flies (Nováková *et al.*^2015^). However, because the genome is in an early draft and has not been released in GenBank, further investigation is necessary to better understand the metabolic roles and the genome evolution of this bacterium.

Recently, the complete genome sequence of an unnamed symbiont (*Arsenophonus* sp. strain CB) of the louse flies *Lipoptena fortisetosa* (Diptera: Hippoboscidae) has been released in GenBank (accession CP013920.1; released on 25 January 2016). Although the biological description and the genome analysis of this organism have not been published yet, its general genomic characteristics (0.8 Mb, 24.9% GC, 74.7% coding, 625 CDSs) and the gene function category distribution are highly similar to those observed among obligate mutualists with highly reduced genomes (Table 1 and Fig. 2). Taken together, these case studies indicated that various lineages within this genus could adopt diverse ecological niches and diverge extensively in their genome characteristics.

### Wolbachia

Bacteria in the genus *Wolbachia* are known for their roles in the manipulation of invertebrate host reproduction (Werren, Baldo and Clark 2008; Saridaki and Bourtzis 2010). Interestingly, while the *Wolbachia* lineages infecting various filarial nematodes are mutualists that codiversify with their hosts, the arthropod-associated lineages are mainly reproductive parasites that show frequent host switches over their evolutionary history. As reproductive parasites, their effects on the hosts include male killing, cytoplasmic incompatibility, parthenogenesis and feminization of genetic males. Genome survey among the insect-associated *Wolbachia* revealed extensive variation in genome size, coding density and gene content (Wu *et al.*^2004^; Klasson *et al.*^2008^, 2009b; Duplouy *et al.*^2013^; Ellegaard *et al.*^2013^; Woolfit *et al.*^2013^; Metcalf *et al.*^2014^; Nikoh *et al.*^2014^; Sutton *et al.*^2014^). The highly dynamic patterns of genome evolution in these bacteria have been linked to the proliferation of mobile genetic elements and extensive intraspecies recombination. Moreover, despite their intracellular lifestyle, which presumably reduced the chance of contact with other bacteria, horizontal gene acquisitions from diverse sources have been reported (Duplouy *et al.*^2013^).

Although horizontally acquired genes generally do not seem to have functional significance and often fail to persist, in some rare cases the acquisition of novel genes could allow the host to exploit novel ecological niches (Kuo and Ochman 2009c). While acquisitions of pathogenicity islands or antibiotic-resistance genes represent some of the best-known success stories, the *Wolbachia w*Cle associated with the bedbug *Cimex lectularius* (Hemiptera: Cimicidae) is an interesting case of parasite-to-mutualist transition mediated by horizontal gene transfer (Hosokawa *et al.*2010b; Nikoh *et al.*^2014^). In this case, the *Wolbachia* is a bacteriocyte-associated endosymbiont that is vertically transmitted. Because the elimination of this *Wolbachia* results in retarded growth and sterility of the host, and the defect could be rescued through oral supplementation of B vitamins, the association appears to be a nutritional mutualist. Through genome analysis, *w*Cle was found to have acquired an operon encoding the complete biotin synthetic pathway, presumably from a co-infecting endosymbiont *Cardinium* or *Rickettsia*. Because of the extensive genome rearrangements observed among *Wolbachia*, it is difficult to infer the exact genomic changes following this ecological transition. However, based on its general genomic features (Table 1) and gene content (Fig. 2), *w*Cle is more similar to parasitic *Wolbachia* than other obligate mutualists, suggesting that the change may have occurred recently.

In addition to *w*Cle, other cases of transition to mutualists such as protection against viruses (Hedges *et al.*^2008^; Teixeira, Ferreira and Ashburner 2008) or increase of host fecundity (Weeks *et al.*^2007^) have been reported, although the genetic mechanisms are less understood. The latter case is of particular interest because the transition from a parasite that reduces the host (i.e. *Drosophila simulans*) fecundity by 15%–20% to a mutualist that increase the host fecundity by 10% has occurred over a period of 20 years, indicating that such ecological and evolutionary transition could occur rapidly.

Another interesting aspect of horizontal gene transfer in *Wolbachia* is that the direction of flow could also go from the symbionts to their hosts (Kondo *et al.*^2002^; Dunning Hotopp *et al.*^2007^; Nikoh *et al.*^2008^; Klasson *et al.*2009a; Hou *et al.*^2014^), a pattern that is parallel to the evolution of mitochondria and plastids (Brown 2003; Keeling, McCutcheon and Doolittle 2015). The sizes of these transferred segments range from few hundred base pairs to nearly the entire *Wolbachia* genome. While most of the transferred genes appeared to have become degraded soon (Dunning Hotopp *et al*^2007^; Nikoh *et al.*^2008^), some were found to have persisted and maintained their expression (Klasson *et al.*2009a). It is possible that these symbiont-to-host gene transfers could be incorporated into the maintenance of symbionts, allowing for further reduction of the symbiont genomes. In addition, cases of mosquito-to-*Wolbachia* gene transfers have also been reported (Woolfit *et al.*^2009^), further demonstrating the role of horizontal gene transfer in promoting evolutionary innovation.

### Spiroplasma

**Figure 3.**
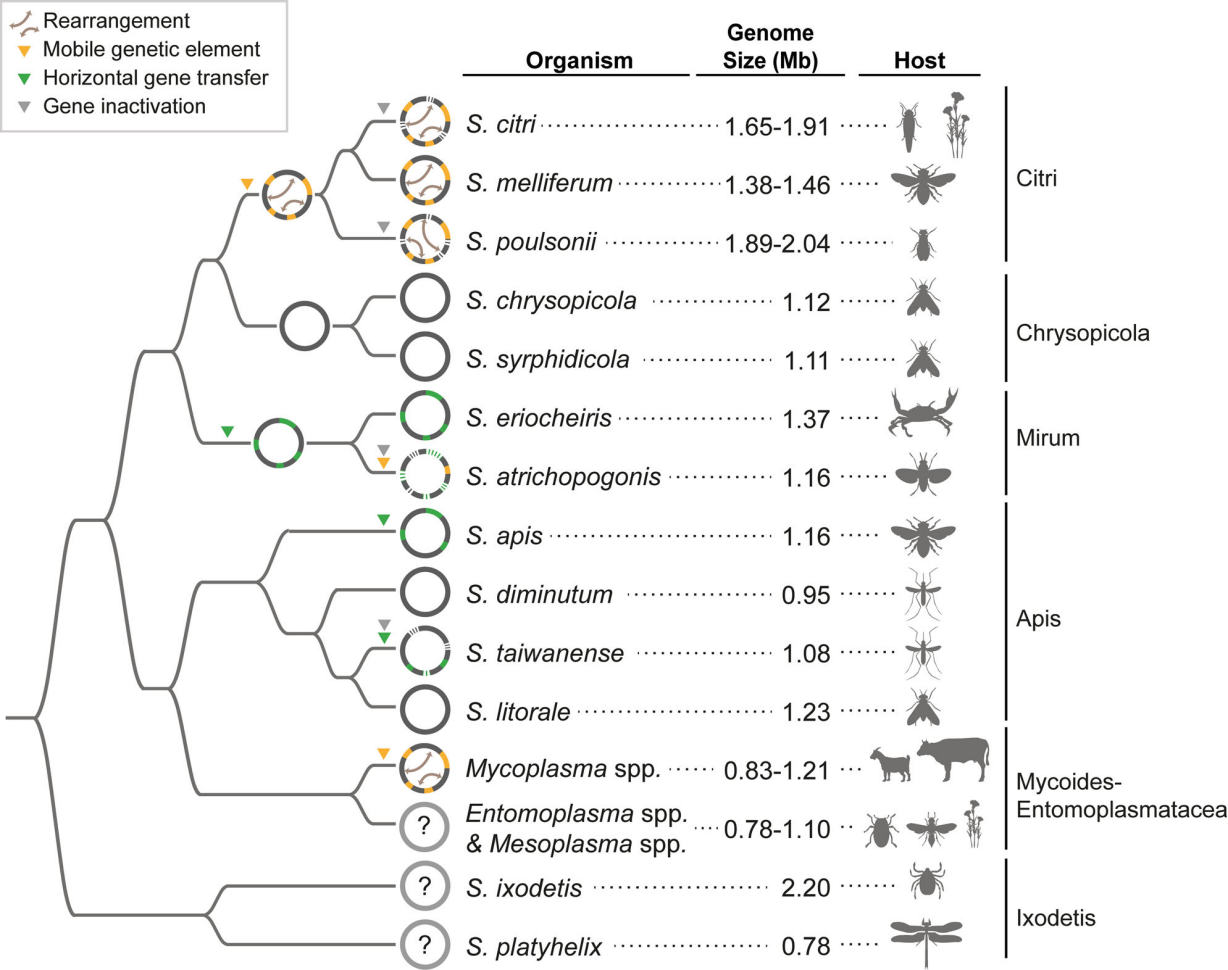
A graphical summary of the genome evolution events in *Spiroplasma* and its derived lineages. Based on the results reported in the literature (Carle *et al.*^2010^; Thiaucourt *et al.*^2011^; Alexeev *et al.*^2012^; Ku *et al.*^2013^; Lo *et al.*2013a,b, 2015; Chang *et al.*^2014^; Ku *et al.*^2014^; Lo, Gasparich and Kuo 2015; Paredes *et al.*^2015^).

The genus *Spiroplasma* contains diverse lineages primarily described as insect associated (Whitcomb 1981; Gasparich *et al.*^2004^; Regassa and Gasparich 2006; Gasparich 2010). Together with the animal pathogens *Mycoplasma* and the insect-transmitted plant pathogens ‘*Ca*. Phytoplasma’, these wall-less bacteria belong to the class Mollicutes, which is related to the Gram-positive bacteria belonging to the phylum Firmicutes (Chen *et al.*^2012^). Ecologically, the *Spiroplasma* species reported to date include harmless commensals, beneficial symbionts, male-killing reproductive parasites and pathogens with varying levels of host dependence. Phylogenetically, *Spiroplasma* and its derived lineages are classified into four major clades (Fig. 3). Among these, three clades are composed of formally described species in the genus *Spiroplasma* (i.e. Citri-Chrysopicola-Mirum, Apis and Ixodetis). The remaining Mycoides-Entomoplasmataceae clade contains a collection of species assigned to the genera *Mycoplasma*, *Mesoplasma* and *Entomoplasma*. Although these nomenclatures create confusions from an evolutionary perspective, the paraphyly of *Spiroplasma* and the polyphyly of *Mycoplasma* have their historical reasons and are unlikely to change due to practical considerations (Gasparich *et al.*^2004^).

In addition to their diverse types of association with a wide range of insect hosts, several other features made *Spiroplasma* a good system to study bacteria–insect symbiosis. First, traits such as male killing or association with specific hosts (e.g. *Drosophila*, honeybee or mosquitos) have arisen multiple times within the genus, which provides excellent opportunities for comparative analysis (Gasparich *et al.*^2004^; Gasparich 2010). At the population level, the ecology of host–symbiont and symbiont–symbiont interactions has received much attention (Anbutsu and Fukatsu 2003; Goto, Anbutsu and Fukatsu 2006; Kageyama *et al.*^2006^; Jaenike *et al.*^2007^; Anbutsu, Goto and Fukatsu 2008; Kageyama *et al.*^2009^; Watts *et al.*^2009^; Toju and Fukatsu 2011; Haselkorn *et al.*^2013^; Haselkorn and Jaenike 2015). Furthermore, unlike many other symbionts that are often difficult (if not impossible) to be cultivated in artificial media outside of their hosts, a large selection of media have been developed early on for cultivating a large number (although not all) of *Spiroplasma* lineages (Chang and Chen 1983; Moulder, French and Chang 2002). Moreover, genetic manipulation has been shown to be feasible in at least one species (Foissac *et al.*^1997^; Killiny *et al.*^2006^). These features, together with the feasibility of using *Drosophila* as a host, make *Spiroplasma* a promising system for investigating the molecular mechanisms of insect–symbiont interactions (Haselkorn, Markow and Moran 2009; Anbutsu and Fukatsu 2011; Herren *et al.*^2013^; Harumoto, Anbutsu and Fukatsu 2014). Finally, the studies on *Spiroplasma* genome evolution also flourished in recent years due to the extensive taxon sampling of available genome sequences. Among the 38 described species, 17 have draft or complete genome sequences available (Carle *et al.*^2010^; Alexeev *et al.*^2012^; Ku *et al.*^2013^, ^2014^; Lo *et al.*2013a,b, 2015; Chang *et al.*^2014^; Davis *et al.*2015a,b; Lo, Gasparich and Kuo 2015; Lo, Liu and Kuo 2015; Paredes *et al.*^2015^). These advancements in genomics have improved our understanding of *Spiroplasma* metabolism and virulence (Bolaños, Servín-Garcidueñas and Martínez-Romero 2015).

Interestingly, as the taxon sampling of available *Spiroplasma* genomes improved over the past five years, it became apparent that the patterns of genome organization and evolution varied considerably across different clades within the genus. For this reason, the following discussion of case studies is organized by clade (Fig. 3).

### *Spiroplasma*: the Citri clade

Within the genus, the Citri clade has received the most attention because it contains the first *Spiroplasma* species to be studied and several economically important pathogens. For example, the type species of the genus, *Spiroplasma citri* (Saglio *et al.*^1973^), is the causative agent of citrus stubborn disease and also the first species to have a draft genome sequence available (Saillard *et al.*^2008^; Carle *et al.*^2010^). *Spiroplasma citri*, the honeybee pathogen *Sp. melliferum* (Alexeev *et al.*^2012^; Lo *et al.*2013a) and the *Drosophila* son-killer *Sp. poulsonii* (Paredes *et al.*^2015^) were all found to harbor abundant prophage sequences in their genomes. These plectroviral sequences account for ∼20% of their chromosomes and represent a daunting challenge in genome sequencing. Even with the help of physical maps and early PacBio sequencing technology, none of these genomes was sequenced to completion. With the improvement in PacBio technology, *Sp. kunkelii* became the first representative of the Citri clade to have the complete genome sequence available in the late 2015, which was also found to harbor these plectroviral sequences (Davis *et al.*2015a).

Extensive proliferation of selfish mobile genetic elements (e.g. insertion sequences, transposons and phages) was thought to be associated with a recent transition to host-restricted lifestyles (Ochman and Davalos 2006; Moran, McCutcheon and Nakabachi 2008). Although this link is probably true for the aforementioned Enterobacteriaceae examples, it does not fit our understanding of *Spiroplasma* biology because such transition probably occurred long ago in the common ancestor of the class Mollicutes and the plectroviral invasion appeared to have originated relatively recent in the common ancestor of the Citri clade (Ku *et al.*^2013^; Paredes *et al.*^2015^). Nonetheless, the extensive proliferation of plectroviral sequences made genome evolution within the Citri clade highly dynamic. For example, *Sp. citri* and *Sp. melliferum* have a genome-wide nucleotide sequence identity of 99%, yet extensive rearrangements were found in their genome alignment (Lo *et al.*2013a). Moreover, general genomic characteristics such as chromosome size, GC content, coding density and gene number all varied considerable among these closely related species (Table 1). Even at the within-species level, strains of *Sp. citri* have been shown to have a genome size range of 1.65–1.91 Mb (Ye *et al.*^1995^), presumably due to variation in plectroviral sequence abundance (Melcher *et al.*^1999^). Similar observations of within-species genome size variation were found in other Citri clade species as well: *Sp. melliferum* has a range of 1.38–1.46 Mb (Carle *et al.*^1995^; Alexeev *et al.*^2012^; Lo *et al.*2013a), *Sp. kunkelii* 1.46–1.61 Mb (Carle *et al.*^1995^; Davis *et al.*2015a) and *Sp. poulsonii* 1.89–2.04 Mb (Williamson *et al.*^1999^; Paredes *et al.*^2015^). In terms of gene content, these species were found to differ substantially in their carbohydrate utilization genes (Carle *et al.*^2010^; Lo *et al.*2013a; Paredes *et al.*^2015^). These differences may be caused by differential gene loss, as well phage-mediated horizontal gene acquisition (Ku *et al.*^2013^), and may have promoted their ecological diversification (Lo *et al.*2013a; Paredes *et al.*^2015^).

Other than the viral invasion and differentiation in gene content, two observations are worth mentioning. First, extensive gene inactivation was found in *Sp. citri* (Carle *et al.*^2010^; Lo *et al.*2013a). In addition to the 1504 full-length CDSs annotated, there are 401 putative pseudogenes annotated as truncated CDSs. Moreover, many of the annotated CDSs could not be assigned to any functional category (Fig. 2), suggesting that a large proportion of them may be fragments of unrecognized pseudogenes. This high level of genome degradation was not found in the closely related *Sp. melliferum* (Alexeev *et al.*^2012^; Lo *et al.*2013a) and the explanation for this difference is unclear. Second, *Sp. poulsonii* was found to contain many more CDSs than other related species (i.e. 2129 compared to 1000–1500). Among these, 523 (24.6%) belong to four families of putative transposases. Unlike the previous discussion on plectrovirus invasion, this proliferation of transposases, as well as a reduction in coding density, is consistent with the expectation that *Sp. poulsonii* has experienced a recent increase in the level of genetic drift (Ochman and Davalos 2006; Moran, McCutcheon and Nakabachi 2008). Indeed, based on the phylogeny and the lifestyle of related species (mostly being able to be transmitted horizontally among hosts), *Sp. poulsonii* became a vertically transmitted reproductive parasite of *Drosophila* only recently. Undoubtedly, such further host restriction would result in a reduction in its effective population size and increase the level of genetic drift.

### *Spiroplasma*: the Chrysopicola clade

In contrast to the highly dynamic patterns observed in the Citri clade, the genome evolution in the Chrysopicola clade is surprisingly static. Currently, the Chrysopicola clade contains two described species associated with hosts from different insect families: *Sp. chrysopicola* (Whitcomb *et al.*^1997^) associated with the deerfly *Chrysops* sp. (Diptera: Tabanidae) and *Sp. syrphidicola* (Whitcomb *et al.*^1996^) associated with the syrphid fly *Eristalis arbustorum* (Diptera: Syrphidae). In 2013, these two species became the first *Spiroplasma* species to have complete genome sequences available, as well as the first ones outside of the Citri clade to be studied (Ku *et al.*^2013^). Notably, there was no evidence of viral invasion ever occurred in the recent evolutionary history of this clade, which is congruent with the existence of intact antiviral systems in these genomes. As a result, genome characteristics such as chromosome size, GC content, coding density, CDS count and gene content are all highly conserved between *Sp. chrysopicola* and *Sp. syrphidicola*. Moreover, despite having a genome-wide nucleotide identity of only 92.2%, a value that is much lower than the 99.0% identity observed between *Sp. citri* and *Sp. melliferum* (Lo *et al.*2013a), only one rearrangement event involving the translocation of a 41-kb segment was found between *Sp. chrysopicola and Sp. syrphidicola*. Because of this chromosomal stability, these Chrysopicola clade genomes served as useful references for inferring the exact boundaries of the plectroviral insertion sites in the Citri clade genomes, and revealed that those phages could mediate horizontal gene transfer among diverse bacteria sharing similar niches (Ku *et al.*^2013^). Unfortunately, the ecology of these two *Spiroplasma* species and their effect on infected hosts has not been studied, and the link between their life history and genome stability is unclear.

### *Spiroplasma*: the Mirum clade

Compared to the viral invasion found in the Citri clade and the stasis found in the Chrysopicola clade, the genome evolution in the Mirum clade is dominated by massive gains (and losses) of horizontally transferred genes (Lo, Gasparich and Kuo 2015). While most described *Spiroplasma* species are associated with terrestrial insects, *Sp. eriocheiris* is a newly emerged lethal pathogen of freshwater crustaceans (i.e. the Chinese mitten crab *Eriocheir sinensis*) (Wang *et al.*^2011^). The *Sp. eriocheiris* genome analysis suggests that ∼7% of its CDSs may have been acquired from lineages outside of *Spiroplasma* and these foreign genes expanded its metabolic capacity (Lo, Gasparich and Kuo 2015). Interestingly, examination of its sister species *Sp. atrichopogonis* (Koerber *et al.*^2005^), which is associated with the biting midges *Atrichopogon* spp. (Diptera: Ceratopogonidae), revealed that the massive gains of novel genes have occurred prior the divergence of these two Mirum clade species. However, in *Sp. atrichopogonis*, most of these horizontally acquired genes have been pseudogenized through accumulation of small deletions or lost entirely. As a result of such different fates for horizontally acquired genes, *Sp. eriocheiris* and *Sp. atrichopogonis*, differ considerably in their chromosome size (1.37 and 1.16 Mb), coding density (86.0% and 71.5%) and full-length CDS count (1180 and 996), while the GC content remains similar (29.8% and 29.3%).

The persistence of these acquired genes in *Sp. eriocheiris* hinted that they might be involved in the ecological shift of this bacterium. In contrast, these novel genes probably did not contribute to the fitness of *Sp. atrichopogonis*, which has maintained its association with terrestrial insects, such that the process of pseudogenization was not countered by selection. However, it is worth noting that psueodgenization is not the only process at work in the evolution of *Sp. atrichopogonis*. After the divergence, *Sp. atrichopogonis* has acquired several transposon-like segments, possibly from Citri-clade donors, indicating frequent genetic exchange between symbionts sharing similar niches, particularly for symbionts with close phylogenetic relationships (Lo, Gasparich and Kuo 2015).

### *Spiroplasma*: the Apis clade

Apis is the most species-rich clade within *Spiroplasma*, containing 23 of the 38 described species (Gasparich *et al.*^2004^). However, the ecology of these species has not been as well studied as those in the Citri or the Mirum clade, possibly reflecting their lack of economical importance. Most work in this clade, including phenotypic assays and genomic characterization, has focused on four mosquito-associated species due to public health concerns (Lo *et al.*2013b; Chang *et al.*^2014^). Through artificial infection experiments (Chastel and Humphery-Smith 1991; Humphery-Smith, Grulet and Chastel 1991; Vazeille-Falcoz, Perchec-Merien and Rodhain 1994; Phillips and Humphery-Smith 1995), it has been established that *Sp. culicicola* and *Sp. taiwanense* are pathogenic, while *Sp. diminutum* and *Sp. sabaudiense* are not. Interestingly, the four species do not form a monophyletic group within the Apis clade (Gasparich *et al.*^2004^; Lo *et al.*2013b), suggesting that the association with mosquito hosts has developed independently. Moreover, the non-pathogenicity observed in *Sp. diminutum* and *Sp. sabaudiense* appears to be resulting from independent losses of the putative pathogenicity factors (Chang *et al.*^2014^). In terms of genome organization, these four species are similar in their chromosome size (ranging from 0.95 to 1.18 Mb). While the genome-wide nucleotide identity ranges from 65.2% to 76.6% in pairwise comparisons, the genome alignments showed that the chromosomal organization is largely conserved. Comparisons of gene content suggest high similarities among these four species, although the carbohydrate utilization genes exhibit complex patterns of presence/absence (Lo *et al.*2013b; Chang *et al.*^2014^). Interestingly, the glycerol uptake and utilization genes that are linked to their pathogenicity in *Sp. taiwanense* are putatively acquired from a Mycoides-Entomoplasmatacea clade donor, while the homologs in *Sp. culicicola* appear to be retained from the ancestor (Chang *et al.*^2014^). *Sp. taiwanense* also suffered degradation in its DNA repair systems and has a much lower coding density (82.5% compared to 90.0%–92.7% in the other three). This observation may be explained by its narrower host range (at least compared to the sympatric *Sp. diminutum*), resulting in a smaller effective population size and elevated genetic drift (Lo *et al.*2013b).

Other than these mosquito-associated species, *Sp. apis* is a species within this clade with more information available. Similar to *Sp. melliferum* in the Citri clade (Clark *et al.*^1985^), *Sp. apis* is known as a honeybee pathogen (Mouches *et al.*^1983^). However, the associations with honeybees in these two species appear to have arisen independently (Gasparich *et al.*^2004^; Lo *et al.*2013a, b). Genome analysis of *Sp. apis* indicated horizontal gene acquisition from divergent sources, but the functional significance is unclear (Ku *et al.*^2014^; Lo, Gasparich and Kuo 2015).

### *Spiroplasma*: the derived Mycoides-Entomoplasmatacea clade

All lineages in the Mycoides-Entomoplasmatacea clade have lost the characteristic helical cell morphology found in their *Spiroplasma* ancestor (Tully *et al.*^1993^; Gasparich *et al.*^2004^), presumably due to the loss of several cytoskeleton genes in the early evolutionary history of this clade (Ku, Lo and Kuo 2014). Within the family Entomoplasmatacea, species are assigned to *Mesoplasma* or *Entomoplasma* based on their sterol requirement. However, the two genera are both polyphyletic and share similar ecology in terms of association with various insect hosts and presence on plant surfaces, suggesting that the lineages in this family could be considered as one coherent group (Gasparich *et al.*^2004^). Several complete or draft genome sequences from this clade have been deposited in GenBank, mostly by the Joint Genome Institute (Department of Energy, USA) through their effort to increase the phylogenetic diversity of available bacterial genomes (Kyrpides *et al.*^2014^). The genome sizes in this group have a range of 0.78–1.10 Mb based on the available data sets, which is similar to those found in the Apis clade (Fig. 3). However, a more detailed analysis of these genomes has not been published yet.

In contrast to its relatives, lineages in the *Mycoplasma* Mycoides clade are not insect associated and have become pathogens of ruminants (Gasparich *et al.*^2004^). The available sequences from this group indicate a genome size range of 0.83–1.21 Mb (Fig. 3). Comparative analysis between two *Mycoplasma mycoides* genomes revealed proliferation of mobile genetic elements, which led to genome rearrangements and degradation (Westberg *et al.*^2004^; Thiaucourt *et al.*^2011^).

### *Spiroplasma*: the Ixodetis clade

The Ixodetis clade is the basal group of *Spiroplasma* (Fig. 3). Although the biological diversity of this clade is quite high, including multiple lineages associated with *Drosophila* spp. (Diptera: Drosophilidae) (Haselkorn, Markow and Moran 2009), an endosymbiont of the chestnut weevil *Curculio sikkimensis* (Coleoptera: Curculionidae) exhibiting infection frequency variation with respect to host plants (Toju and Fukatsu 2011) and a male killer of the ladybird beetle *Anisosticta novemdecimpunctata* (Coleoptera: Coccinellidae) (Tinsley and Majerus 2006), most of these lineages have not been isolated and formally described. Currently, this clade contains only two described species: *Sp. ixodetis* associated with the tick *Ixodes pacificus* (Tully *et al.*^1995^) and *Sp. platyhelix* associated with the dragonfly *Pachydiplax longipennis* (Odonata: Libellulidae) (Williamson *et al.*^1997^). Incidentally, these two species have the largest and the smallest genome reported within the genus, respectively (Fig. 3). Because of this variation in genome size, as well as their phylogenetic placement, future genome characterization effort in this clade could greatly improve our understanding of *Spiroplasma* genome evolution.

## COMPARISONS AMONG FACULTATIVE SYMBIONTS

One of the most fascinating aspects about biology is the extensive diversity observed in nature. Because of this, drawing general patterns of biological systems is often difficult. As noted above, the facultative insect symbionts with a genome size of 1–5 Mb are not really a homogeneous group. Even at the genus level, different clades within *Spiroplasma* clearly exhibit widely different patterns of genome evolution. Moreover, the three major taxonomic groups discussed clearly have different ancestral states, functional roles and evolutionary trajectories. Within Enterobacteriaceae, the symbionts discussed above (i.e. *Pantoea*, *Serratia*, etc.) likely have evolved recently from a non-host-restricted ancestor, which had a genome size of 4–6 Mb and >4000 CDSs. Even with the massive genome reduction, many genes involved in various biosynthesis pathways have been maintained in several cases. While gene inactivation may have disrupted some of the important pathways, such deficiencies could be overcome through horizontal gene acquisition or integration into a tripartite symbiosis. As such, some of these symbionts may (or already have) become nutritional mutualists and likely will continue on the trajectory of further genome reduction (e.g. ‘*Ca*. Pantoea carbekii’ and *Sodalis* sp. PSPU).

In comparison, *Wolbachia* belongs to the family Rickettsiales, which contains two other genera of obligate intracellular parasites (i.e. *Rickettsia* and *Orientia*), thus is likely to have a host-restricted ancestor with a highly reduced genome. Currently, most insect-associated *Wolbachia* have been highly successful as reproductive parasites (Werren, Baldo and Clark 2008; Saridaki and Bourtzis 2010). Although this specialization may represent an evolutionary dead end, the lack of concordance between the phylogeny of *Wolbachia* and that of their hosts indicates that these parasites could escape extinction through frequent host switches. Moreover, the reports of parasite-to-mutualist transitions (Weeks *et al.*^2007^; Hedges *et al.*^2008^; Teixeira, Ferreira and Ashburner 2008; Hosokawa *et al.*2010b; Nikoh *et al.*^2014^) suggest that even for these obligate intracellular parasites with highly reduced genomes, the potential evolutionary trajectories remain surprisingly flexible.

For *Spiroplasma*, inferring the ancestral state is difficult because the related *Mycoplasma* and ‘*Ca*. Phytoplasma’ mostly have more reduced genomes (Chen *et al.*^2012^). With their current gene content, the trajectory of becoming nutritional mutualists seems unlikely. Currently, most of the described *Spiroplasma* lineages live extracellularly inside their insect hosts, while maintaining the ability of horizontal transmission among hosts through survival on plant surfaces (Gasparich *et al.*^2004^; Regassa and Gasparich 2006; Gasparich 2010). Thus, the possible evolutionary trajectories for *Spiroplasma* may be more flexible compared to *Wolbachia* due to better chances of gene acquisition and host switch. Indeed, in addition to becoming vertically transmitted reproductive parasites (Williamson *et al.*^1999^; Tinsley and Majerus 2006), *Spiroplasma* lineages have been found to evolve into beneficial symbionts that provide protection against biotic stresses (Jaenike *et al.*^2010^; Xie, Vilchez and Mateos 2010; Lukasik *et al.*^2013^). Moreover, the Citri clade has been successful in becoming insect-transmitted pathogens of monocotyledon and dicotyledon plants (Gasparich 2010), while at least two independent events have results in host switches to freshwater or marine crustaceans (Nunan *et al.*^2005^; Wang *et al.*^2011^). Finally, in the case of the derived *Mycoplasma* Mycoides clade, the group has become vertebrate pathogens that do not rely on arthropod vectors (Gasparich *et al.*^2004^).

## A MORE DETAILED EVOLUTIONARY MODEL

**Figure 4.**
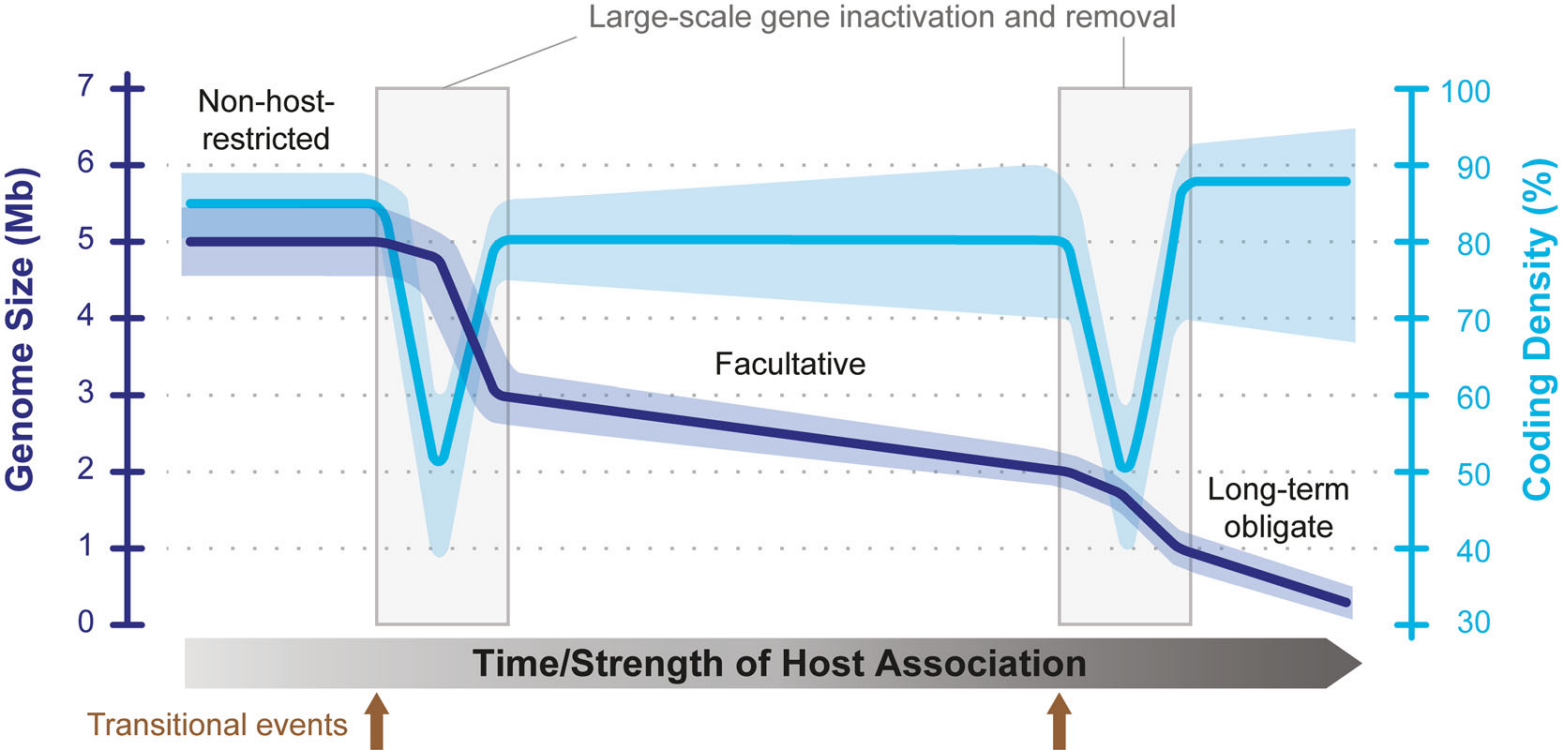
Trajectories of genome size and coding density in symbiont evolution. During the process, one or more transitional events (e.g. strict host association, vertical transmission or endosymbiosis) could cause a sudden reduction in the effective population size and release a substantial proportion of genes from selective constraints. As a result, non-adaptive mutation accumulation could lead to large-scale gene inactivation, lowering the coding density. These pseudogenes would eventually be removed due to the mutational bias toward deletions commonly observed in bacterial genomes, leading to a higher coding density and a much smaller genome. In addition, small-scale events of genome degradation could occur continuously throughout the process. Importantly, not all symbionts would eventually develop obligate symbiosis and have extremely reduced genomes. Some may maintain facultative associations with their hosts and be commensals, parasites or mutualists. Transitions between symbiosis types and host switches may be possible by acquiring new genes through horizontal gene transfer or losing key genes through mutations.

With the aforementioned diversity in mind, here we attempt to incorporate the recent findings discussed above, including several putative ‘missing links’ (Lamelas *et al.*^2011^; Kenyon, Meulia and Sabree 2015; Hosokawa *et al.*^2016^), into the conceptual frameworks established previously (Lynch 2006; Ochman and Davalos 2006; Moran, McCutcheon and Nakabachi 2008; Kuo, Moran and Ochman 2009; Toft and Andersson 2010; McCutcheon and Moran 2012; Moran and Bennett 2014) and provide a more detailed model of symbiont genome evolution (Fig. 4). For the starting point, we envision an Enterobacteriaceae-like non-host-restricted ancestor with a relatively large genome of 4–6 Mb. Prior to the development of an intimate association with its eukaryotic host, small fluctuations in genome size and coding density are expected due to the gene gains and losses occurring constantly (Kuo and Ochman 2009c). As seen in *Sodalis* and *Serratia symbiotica* (Table 1), the development of symbiosis could result in one or more large waves of gene inactivation that highly reduce the coding density. The triggers for such large-scale pseudogenization events may involve substantial changes in the physiological environment or ecology, such as the transition from extracellular to intracellular associations with the host or adopting strict vertical transmissions. The change to a more stable and nutrient-rich environment provided by the host could release a large number of CDSs from selective constraints, while an increase in host restriction would decrease the effective population size. These two factors would lead to rapid and large-scale pseudogenization events, which produce the sudden and deep drops in coding density (e.g. from ∼85%–90% to ∼40%–60%) (Table 1 and Fig. 4).

Because most insertions and deletions observed in the mutational input of bacteria genomes are <100 base pairs (Kuo and Ochman 2009a), a lag time is expected before these newly formed pseudogenes could be removed through either adaptive or non-adaptive processes. After pseudogene removal, the coding density would return to a range of ∼70%–90%, while the genome size becomes substantially smaller prior to these large waves of gene inactivation (Fig. 4). Importantly, the inactivation of CDSs and the subsequent pseudogene removal is a continuous process that could occur in both large and small scales, possibly at an ever-increasing rate as the effective population size continues to drop. As the genome becomes smaller and the number of CDSs decreases, a larger variation in the coding density observed is expected (Table 1 and Fig. 4).

During the initial stages of gene inactivation, the gene loss may be more or less stochastic, rather than deterministic, as the overall functional category distribution of the survived genes found in recently evolved symbionts is not substantially different from their non-host-restricted relatives (Fig. 2). However, the exact genes and metabolic pathways remained in these moderately reduced genomes could greatly influence the subsequent evolutionary trajectory. For example, if the complete gene sets for providing certain benefits to the hosts (e.g. nutrition or stress protection) are disrupted, the symbionts could maintain commensal relationships with their hosts. Alternatively, the symbionts could become specialized in parasitic lifestyles. Depending on the ecology (e.g. a need for survival outside of the host during horizontal transmissions), the process of genome degradation could be slowed down by selection (e.g. *Spiroplasma*). Occasionally, the symbionts may acquire novel genetic materials through horizontal transfers, which allow for host switches or transitions into mutualists [e.g. *Wolbachia w*Cle (Hosokawa *et al.*2010b; Nikoh *et al.*^2014^) and ‘*Ca*. Profftella armatura’ (Nakabachi *et al.*^2013^)]. The invasions of mobile genetic elements (e.g. transposable elements and phages) may promote such gene acquisitions. Thus, the transition of facultative to obligate symbiosis is not necessary a universal one-way street.

If a mutualistic relationship could be formed, selection may then be involved in shaping the symbionts into obligate mutualists (Moran and Bennett 2014). Thus, for the lineages that are in transition from facultative to obligate symbiosis, poorly characterized genes, which may be required under certain specific environmental conditions for their non-host-restricted ancestors but are not essential (Baba *et al.*^2006^), would be further released from selective constraint and lost. As observed in the currently available data sets (Fig. 2), lineages that transited into obligate mutualism recently would have a gene functional category distribution similar to their relatives that still maintain a facultative lifestyle (e.g. ‘*Ca*. Hamitonella defensa’ MEAM1 and *Wolbachia w*Cle), while those representing later stages of the transition (e.g. *S. symbiotica* SCc and ‘*Ca*. Pantoea carbekii’) would have a pattern more similar to obligate mutualists with highly reduced genomes (e.g. *B. aphidicola*).

Eventually, the obligate mutualists could have a highly reduced genome of <1 Mb (Table 1). At this stage, genes required for symbiont survival (e.g. information storage and processing) and maintenance of the mutualistic relationship (e.g. biosynthetic pathways of specific nutrients) would account for the majority of gene content (Fig. 2). However, as the process of genome reduction and degradation continues (e.g. for those ones with a genome size < 0.3 Mb), high levels of nucleotide compositional bias and protein sequence divergence could hinder functional category assignment by standard database searches for a considerable proportion of genes.

Other than genome size and coding density, some other notable changes occurred during the process of genome reduction include decreases in GC content (Table 1) and in some cases, adoption of alternative genetic codes. The trend of GC content evolution has been well documented (McCutcheon and Moran 2012; Nishida 2012). Even though such a trend is likely to be deleterious (Hildebrand, Meyer and Eyre-Walker 2010; Raghavan, Kelkar and Ochman 2012), natural selection is not effective in these symbionts with small effective population sizes. Instead, the strong mutational bias toward AT (Hershberg and Petrov 2010; Hildebrand, Meyer and Eyre-Walker 2010; Van Leuven and McCutcheon 2012; Lassalle *et al.*^2015^) and non-adaptive loss of DNA repair genes (McCutcheon and Moran 2012) are the main driving force. However, it is worth noting that although this trend of GC content reduction is apparent at a large scale, exceptions and more complex patterns at smaller scales do exist. For example, two obligate symbionts with extremely reduced genomes were found to have a relatively high GC content (McCutcheon, McDonald and Moran 2009; McCutcheon and von Dohlen 2011). These outliers are ‘*Ca*. Hodgkinia cicadicola’ (0.14 Mb; 58.4% GC) and ‘*Ca*. Tremblaya princeps’ (0.14 Mb; 58.8% GC). Additionally, for the within-genus comparisons among *Spiroplasma* species, the GC content and genome size do not show significant correlation (Table 1). Further studies are required to investigate these intriguing disparities.

Among the symbiotic bacteria with reduced genomes, the adoption of alternative genetic codes has occurred in *Spiroplasma* (prior to its divergence from other related *Mycoplasma* clades) (Bové 1993), ‘*Ca*. Hodgkinia cicadicola’ (McCutcheon, McDonald and Moran 2009), ‘*Ca*. Zinderia insecticola’ (McCutcheon and Moran 2010) and ‘*Ca*. Nasuia deltocephalinicola’ (Bennett and Moran 2013). A single change from the standard code (i.e. re-assigning ‘UGA’ from stop to tryptophan) was involved in all of these cases. This re-assignment has also occurred independently in several mitochondrial lineages and may be explained by the ‘codon capture’ theory (Osawa and Jukes 1989; Knight, Freeland and Landweber 2001). It is unclear if these stochastic events have any functional consequences. One perceivable effect of adopting an alternative genetic code is that it may impede horizontal gene transfer. Among the cases of inferred horizontal gene acquisitions in *Spiroplasma*, the major sources appear to be those from within the class Mollicutes and share this alternative code (Chang *et al.*^2014^; Lo, Gasparich and Kuo 2015). However, this observation may be explained by their shared ecological niches and codon usage biases, such that gene transfers are facilitated by frequent contacts and possibly higher chances of persistence. Additionally, multiple gene acquisitions from the more distantly related Firmicutes or even Gammaproteobacteria have been found as well (Lo, Gasparich and Kuo 2015), suggesting that this potential barrier may not be strong, if present at all.

## CONCLUDING REMARKS

As typical in scientific endeavors, the more we know, the more questions we have. With the recent progress in characterizing insect gut microbiota, it becomes apparent that diverse bacterial communities could form stable associations with various insects (Moran *et al.*^2012^; Osei-Poku *et al.*^2012^; Engel and Moran 2013; Shelomi *et al.*^2013^; Sabree and Moran 2014; Anderson *et al.*^2016^). In addition to the lineages discussed above, other Proteobacterial classes, Actinobacteria, Bacteroidetes and Firmicutes are often abundant residents in insect guts. Presumably, some (or many) of these diverse bacteria could have developed highly intimate relationships with their insect hosts but are yet to be studied. Given the observation that several obligate mutualists have originated from Betaproteobacteria and Bacteroidetes (Moran, McCutcheon and Nakabachi 2008; Toft and Andersson 2010; McCutcheon and Moran 2012), it is likely that future work could identify more transitional lineages in these phyla. In that case, we could further improve our understanding of symbiont diversity in terms of their evolutionary history and trajectories.

Regarding the functional roles of symbionts, nutrition provision is the predominate type found among the characterized obligate mutualists (Moran, McCutcheon and Nakabachi 2008; McCutcheon and Moran 2012; Moran and Bennett 2014). The reason for this prevalence may be that this function helps to expand the ecological niches of the host, thus promoting codiversification and resulting in more case reports. Moreover, functional characterization of nutritional mutualism may be more straightforward with the research tools currently available. In comparison, other than nutrition provision, several facultative symbionts have been found to provide their hosts with protection against biotic or abiotic stress. Undoubtedly, these types of environment-dependent mutualisms would be more difficult to be noticed and studied, which may result in a sampling bias in our understanding of insect–bacteria symbiosis. In addition to further characterization of these protectional mutualisms, it would be interesting to see what other functional roles could be found among symbiotic bacteria.

Finally, on a higher and perhaps more philosophical level, the study of symbiosis has long been linked to the discussion of what constitutes an individual, as well as the level at which natural selection operates. With the continuous advancement in our understanding of microbiome, the concepts of ‘hologenome’ and ‘holobiont’ certainly warrant further discussion and empirical studies (Zilber-Rosenberg and Rosenberg 2008; Gilbert, Sapp and Tauber 2012; McFall-Ngai *et al.*^2013^; Bordenstein and Theis 2015; Moran and Sloan 2015).

## FUNDING

This work was supported by research grants from the at and the [grant numbers and ] to CHK.

***Conflict of interest.*** None declared.
